# Correlation of lateral stenosis in MRI with symptoms, walking capacity and EMG findings in patients with surgically confirmed lateral lumbar spinal canal stenosis

**DOI:** 10.1186/1471-2474-15-247

**Published:** 2014-07-23

**Authors:** Pekka Kuittinen, Petri Sipola, Timo Juhani Aalto, Sara Määttä, Anita Parviainen, Tapani Saari, Sanna Sinikallio, Sakari Savolainen, Veli Turunen, Heikki Kröger, Olavi Airaksinen, Ville Leinonen

**Affiliations:** 1 Department of Neurosurgery, Kuopio University Hospital, Kuopio, Finland; 2 Department of Clinical Radiology, University of Eastern Finland, Kuopio, Finland; 3 Department of Clinical Radiology, Kuopio University Hospital, Kuopio, Finland; 4 Kyyhkylä Rehabilitation Center and Hospital, Mikkeli, Finland; 5 Department of Clinical Neurophysiology, University of Eastern Finland, Kuopio, Finland; 6 Department of Clinical Neurophysiology, Kuopio University Hospital, Kuopio, Finland; 7 Department of Clinical Neurophysiology, North Karelia Central Hospital, Joensuu, Finland; 8 University of Eastern Finland, Institute of Public Health and Clinical Nutrition, Kuopio, Finland; 9 Department of Orthopedics and Traumatology, Kuopio University Hospital, Kuopio, Finland; 10 Bone and Cartilage Research Unit, University of Eastern Finland, Kuopio, Finland; 11 Department of Physical and Rehabilitation Medicine, Kuopio University Hospital, Kuopio, Finland

## Abstract

**Background:**

To evaluate the clinical significance of lateral lumbar spinal canal stenosis (LLSCS), found by magnetic resonance imaging (MRI), through correlating the imaging findings with patient symptoms, walking capacity and electromyography (EMG) measurements.

**Method:**

102 patients with symptoms of LSS referred for operative treatment were studied in this uncontrolled study. Of these patients, subjects with distinct only lateral LSS were included. Accordingly, 140 roots in 14 patients (mean age 58, range 48-76 years, male 43%) were evaluated. In MR images the entrance and mid zones of the lateral lumbar nerve root canal were graded as normal, narrowed but not compressed, or compressed. In quantitative analysis, the minimal widths of the lateral recess and mid zone area were measured. Clinical symptoms were recorded with the Oswestry Disability Index (ODI), overall Visual Analogue Scale (VAS), specific low back pain (LBP; NRS-11), specific leg pain (LP NRS-11), Beck Depression Inventory (BDI) and walking distance in the treadmill test. Lumbar paraspinal (L2- L5) and lower limb (L3 – S1) needle EMG studies were performed. The findings were classified root by root as 1 = normal, 2 = abnormal. The associations between radiological, EMG and clinical findings were tested with each other.

**Results:**

EMG findings were normal in 92 roots and abnormal in 48 roots. All of the patients had at least one abnormal nerve root finding. Severity of the mid zone stenosis in MRI correlated with abnormal EMG findings (p = 0.015). Patients with abnormal EMG had also higher scores in the VAS (41.9 ± 25.7 vs 31.5 ± 18.1; p = 0.018), NRS leg pain (7.5 ± 1.5 vs 6.3 ± 2.1; p = 0.000) and BDI (9.8 ± 3.8 vs 8.0 ± 3.9; p = 0.014). However, no statistically significant correlations between MRI findings and clinical symptoms or walking capacity were found.

**Conclusions:**

Among persons previously selected for surgery, lateral stenosis seen on MRI correlates with EMG, and thus may be a clinically significant finding. Our EMG findings were also associated with patient symptoms. However, no relationships between the MRI findings and symptoms or walking capacity were found, suggesting their multifactorial etiology.

## Background

Lumbar spinal stenosis (LSS) is defined as "buttock or lower extremity pain, which may occur with or without low back pain, associated with diminished space available for the neural and vascular elements in the lumbar spine" [1]. Lateral lumbar spinal canal stenosis (LLSCS) is a related condition and it is characterized by the narrowing of the lateral aspects of the central canal (subarticular recess) or foramen through which the nerve root exits the spinal canal. LSS is the most common indication for lumbar spinal surgery in people aged over 65 years [2]. When successful, surgery relieves pressure on the nerves and reduces pain and weakness. However, the long-term results of surgery are poor in one third of patients [2,3]. Accordingly, preoperative patient selection is considered critical. Clinically, routine magnetic resonance imaging (MRI) and electromyography (EMG) are standard tools in the diagnostic workup of patients with suspected LSS [4-8].

Most of the earlier studies in patients with LSS are focused on patients with central canal stenosis. LLSCS is a controversial clinical issue. On one hand it is thought that the most common cause for a poor surgical result in LSS surgery was failure of the surgeon to either identify or adequately treat the LLSCS [9]. On the other hand, LLSCS is thought to be over-diagnosed because the pathology can be more readily seen on MRI and CT scans and is also seen in asymptomatic patients.

The purpose of this study was to evaluate correlation of MRI imaging findings, EMG and clinical patient symptoms with LLSCS patients.

## Methods

### Patients

This prospective single center study was approved by the Ethics Committee of Kuopio University Hospital, and the patients provided written informed consent. This study consisted of 102 LSS patients diagnosed by a treating surgeon who reviewed clinical and imaging findings (MRI). Out of the 102 patients, only 14 with distinct only lateral stenosis were included. None of the patients had central canal stenosis. Accordingly, 140 roots of the 14 patients (mean age 58, range 48 - 76 years, male 43%) were assessed. Initial radiological evaluation was made by a neuroradiologist with 15 years of experience (TS). Selection for surgery was made by an orthopedic surgeon or neurosurgeon in Kuopio University Hospital, Kuopio, Finland. Potential subjects who were not offered surgery were not included.

The exclusion criteria were: emergency or urgent spinal surgery precluding recruitment and protocol investigations; cognitive impairment prohibiting completion of the questionnaires or other failures in co-operation, and the presence of metallic objects in the body that prevented magnetic resonance imaging. A coexisting disc herniation was not an exclusion criterion, but the primary diagnosis of the study patients had to be LLSCS.

The inclusion criteria were: 1) the presence of severe back, buttock, and/or lower extremity pain and/or neurogenic claudication with radiographic evidence (magnetic resonance imaging) exiting nerve roots by degenerative changes (ligamentum flavum, facet joints, osteophytes, and/or disc material), and 2) the surgeon’s judgment in clinical and radiological evaluation that the patient had degenerative LSSCS with symptoms that could be relieved by operative treatment. In addition, all patients had a history of ineffective response to conservative treatment over three months. Patients with only back pain were not included.

### Magnetic resonance imaging

MR imaging of the lumbar spine was performed with a 1.5 T imager (Vision; Siemens Medical Solutions, Erlangen, Germany) and a dedicated receive-only spine coil. All patients were imaged prospectively with the same study protocol for study purposes. The imaging protocol conformed to the requirements of the American College of Radiology for the performance of MRI of the adult spine [10]. The following sequences were used: (a) sagittal T1-weighted spin-echo (repetition time/echo time (TR/TE) 600/12 ms; flip angle, 150°; 4 mm sections; intersection gap, 0.4 mm; field of view (FOV), 290 mm; rectangular FOV, 80%; three signals acquired per data line; matrix 288 × 512), (b) sagittal T2-weighted fast spin-echo (3500/120; flip angle, 180°; echo train length of five; 4 mm sections; intersection gap, 0.4 mm; FOV 290 mm; rectangular FOV, 63%; two signals acquired; matrix 180 × 512), (c) transverse T1-weighted spin-echo (700/15; flip angle, 90°; 4 mm sections; intersection gap, 0.4 mm; FOV, 250 mm; rectangular FOV, 80%; two signals acquired per data line; matrix 288 × 512), and (d) transverse T2-weighted fast spin-echo (5000/120; flip angle, 180°; echo train length of 15; 4 mm sections; intersection gap, 0.4 mm; FOV, 250 mm; rectangular FOV, 100%; three signals acquired per data line; matrix 330 × 512).

The entire lumbar spine was studied on the sagittal images (T12-S1) including parasagittal imaging of all the neural foraminae bilaterally. Transverse images were obtained from the inferior aspect of L1 to the inferior aspect of S1, and the orientation of the sections was planned parallel to the major axis of each disc. In all sequences, a saturation band was placed over the abdominal vessels.

### Image analysis

Image analysis was performed as previously described in detail [11]. Briefly, the lateral canal of the lumbar spine was divided into subarticular (entrance) and foraminal (mid) zones. The subarticular zone (lateral recess) was the most cephalad part of the lateral lumbar canal and located medial to or underneath the superior articular process. The foraminal zone was located below the pedicle. Each subarticular zone and foraminal zone was evaluated separately, bilaterally. The observer was blinded to the clinical and radiological reports and to the findings of any prior clinical examinations. The observer was, however, aware that all study subjects were symptomatic. Visual assessment and quantitative measurements were performed on an IDS5 diagnostic workstation (version 10.2P4; Sectra Imtec, Linköping, Sweden) using highly magnified images on 1024 × 768 and 1600 × 1200 displays.

In visual analysis, the grading system classified the lumbar nerve root canals into three grades: 0 = normal, 1 = narrowing without root compression and 2 = nerve root compression. In quantitative analysis, the minimal width of the subarticular (entrance) zone (lateral recess) and the cross-sectional (mm^2^) area of the foraminal zone (mid zone area) were measured. At the foraminal zone, no space below the line parallel to the lower end plate was included in area measurements as previously described in detail [11]. Repeatability of assessments has been previously studied and shown to vary from moderate to substantial [11].

### Assessment of preoperative symptoms and functional disability

The overall current low back and leg pain intensity was assessed by a self-administered Visual Analogue Scale (VAS) (range 0-100 mm) in a sitting position during study visits. This has been proved to be a valid index of experimental, clinical and chronic pain [12]. Back pain at rest (during the previous week) and leg pain when walking (during the previous week) were measured separately with a numeric rating scale 0-10 (NRS-11) [13]. The questions about pain were anchored on the left (0) with the phrase "No pain" and on the right (10) with the phrase "intolerable pain".

Subjective disability was measured by the validated Finnish version of the Oswestry Disability Index, where 0% represents no disability and 100% extreme debilitating disability [14-16].

Depression was assessed with the Finnish version of the 21-item Beck Depression Inventory (BDI) with scores ranging from 0 to 63 [17,18].

The treadmill test was supervised by a physiotherapist. The patient was asked to keep a straight, upright position during walking (with a zero degree ramp). The starting speed was 0.67 m/s for the first 10 min (400 m), then 1 m/s for the next 10 min (600 m); maximum results were thus 1000 m in 20 min. If the patient was not able to start with a speed of 0.67 m/s, another test with a starting speed of 0.5 m/s was applied. Thus the walking distance scale was 0-1000 m.

### EMG

Lumbar paraspinal and lower limb needle EMG were recorded pre-operatively by a neurophysiologist (SM or AP) who was blinded to the radiological data and clinical assessment. The EMG investigation included bilateral paraspinal muscles innervated by the L2-L5 posterior primary rami and a symptomatic lower limb muscles (roots L3 - S1). Examination of the paraspinal roots was performed using a monopolar needle electrode (Medtronic, 50 × 0.40 mm) and examination of the lower limb muscles using a concentric (Neuroline, 38 × 0.45 mm) needle electrode. Amplification was set at 50 μV/div, and the high and low-pass filters at 10 kHz and 20 Hz, respectively.

The aim of the needle examination was to detect the abnormal spontaneous activity associated with axonal damage (fibrillation and positive sharp waves) [8]. Our EMG data was scaled in the following way: 0 = none or no reproducible spontaneous activity, 1 = rare or occasional (two or more) trains of fibrillation potentials, 2 = frequent spontaneous potentials recordable at more than one depth, 3 = abundant spontaneous activity nearly filling the screen. Categories 1-3 were considered abnormal.

During paraspinal EMG, the patient would lie in the prone position supported by pillows underneath the abdomen. The L3-4 interspinal space was determined by first locating the interspinal space at the level corresponding to the iliac crest, then identifying the L3, L4, L5 and S1 spinal processes by palpation. At least 20 insertions were analyzed from each multifidus muscle. The aim of the examination was to detect the abnormal spontaneous activity suggesting lower motor neuron disorder, and thus serving as a sign of denervation. Abnormal activity indicating denervation was considered to be fibrillation potentials, positive sharp waves, and complex repetitive discharges.

Lower limb needle EMG was performed with the patient lying in the supine position, considering assessment of m. vastus lateralis, tibialis anterior, extensor hallucis longus and gastrocnemius (roots L3 -S1).

The recording of tibial H-waves and peroneal F-waves was performed with Keypoint EMG equipment (Skovlunde, Denmark), using the H-wave and F-wave programs (20 successive samples, 1 stimulus per second, stimulus duration 0.2 ms, high pass filter 20 Hz, low pass filter 10 kHz, sensitivity 0.2 mV/div, sweep 10 ms/div). The stimulus intensity for F-responses was adjusted to obtain supramaximal M-response amplitude. The F-responses were recorded using a surface electrode placed over the middle of the short toe extensor muscle. The reference electrode was placed over the first metatarsal bone on the dorsal surface of the foot. The stimulus intensity for H-responses was adjusted to get repeatable responses with identical latency. The stimulus site was the popliteal fossa and the H-responses were recorded by surface electrodes over the gastrocnemius muscle. The latencies of the minimum F-response (Fmin) and H-response were determined. The recorded values were compared to the normal material of the laboratory (120 normal values) taking the height of the subject into account. The deviation from the normal value was expressed as standard deviation (SD). SD values higher than 2.5 SD were considered abnormal.

Nerve root level specific (L2 – S1) EMG was abnormal if abnormal spontaneous activity associated with axonal damage (fibrillation and positive sharp waves) was found in the limbs or paraspinals. Specific nerve root involvement was defined by that nerve root innervated paraspinal muscles (roots L2 – L5) or lower limb muscles (vastus lateralis, tibialis anterior, extensor hallucis longus and gastrocnemius) (roots L3 – S1). The EMG findings were classified root by root as 1) normal (92/140 roots) or 2) abnormal (active paraspinal and/or limb lesion (48/140).

### Statistical analyses

In statistical analyses, MRI and EMG were analyzed root by root. Associations between MRI findings with VAS, BDI, walking capacity and EMG were analyzed using Chi-squared tests, Spearman and Pearson correlation coefficients, t-tests, and when no assumption of normal distribution could be made, non-parametric tests were used. Statistical analysis was performed using SPSS for Windows (version 19.0; SPSS, IBM, Chicago IL, USA). Statistical significance was set at the P < 0.05 level.

## Results

Patient characteristics are summarized in Table 1. The mean age of the study patients (n = 14) at the time of surgery was 58 years (range 48-76), and 6 (42%) of the subjects were male. None of the patients had undergone previous spine surgery. Co-existing disc herniation was found in one patient (7%).

**Table 1:** Clinical characteristics of the study subjects with lateral spinal stenosis (n = 14 patients)

Male/Female	6/8 (43/57)
Marital status; married or co-habiting	9 (64)
Current smoker	2 (14)
Age	58 (range 48-76)
BMI (kg/m^2^)	27.3 (3.8)
Number of somatic diseases	5.8 (3.9)
ODI	41.5 (9.6)
VAS overall	35.1 (22.2)
NRS LBP	4.4 (3.0)
NRS LP	6.7 (2.1)
BDI score	8.6 (4.1)
Walking distance (m)	829 (310)

Note: Except where indicated, data are numbers of patients, with percentages in parentheses or means, ± standard deviations in parentheses.

ODI = Oswestry disability index scale (0-100), VAS overall = Visual analogue pain scale (0-100), NRS LBP = NRS low back pain at rest, scale (0-10), NRS LP = NRS leg pain at walking, scale (0-10), BDI = Beck Depression inventory (0-63).

According to the ODI scores, 1 patient (7%) had minimal disability (scores 0-20), 4 patients (29%) had moderate disability (scores 21-40), 9 patients (64%) had severe disability (scores 41-60), and none of the patients were crippled (scores over 60). In the overall VAS scores, 3 patients (21%) had minimal pain (scores 0-20), 7 (50%) had moderate pain (scores 21-40), 2 (14%) had severe pain (scores 41-60) and 2 (14%) had crippling pain (scores >60). In the BDI scale, 12 patients had normal mood and 2 patients were depressed (15 or more points), with a mean BDI of 8.6 (SD 4.1) and the walking distance was 829 ± 310 meters (Table 1).

By MRI, the lateral spinal canal recess appeared normal in 93 roots, narrowed in 35 roots and compressed in 12 roots. Also according to our MRI findings, the lateral foraminal canal was normal in 111 roots, narrowed in 26 roots and compressed in 3 roots. The mean entrance zone width of the lateral spinal canal was 5.3 (1.8) mm and the foraminal zone area was 69.3 (17.6) mm^2^, respectively.

The EMG findings were normal in 92 (66%) roots and abnormal in 48 (34%) roots. Level by level EMG data were as follows: L2; 27 roots normal and 1 root abnormal, L3; 11 roots normal and 17 roots abnormal, L4; 19 roots normal and 9 roots abnormal, L5; 14 roots normal and 14 roots abnormal, S1; 21 roots normal and 7 roots abnormal.

F-responses were bilaterally normal in 4 patients, one-side were normal and one-side were abnormal in 5 patients and bilateral abnormal F-responses were in 5 patients, respectively. H-responses were bilaterally normal in 6 patients, one-side were normal and one-side were abnormal in 3 patients and bilateral abnormal H-responses were in 5 patients, respectively.

### Correlation of MRI findings with clinical symptoms

Visually assessed severity of entrance stenosis, mid zone stenosis, entrance zone width, and mid zone area did not correlate with the clinical symptoms recorded; ODI, VAS, specific low back pain (LBP; NRS-11), specific leg pain (LP NRS-11), BDI and walking distance achieved in the treadmill test.

### Correlation of clinical symptoms with EMG findings

Patients with abnormal EMG findings had higher scores in the VAS (41.9 ± 25.7 vs 31.5 ± 18.1; p = 0.018), VAS leg pain (7.5 ± 1.5 vs 6.3 ± 2.1; p = 0.000) and BDI (9.8 ± 3.8 vs 8.0 ± 3.9; p = 0.014) (Table 2).

**Table 2:** Comparison of normal and abnormal EMG groups with clinical data (n = 14)

	**Normal EMG**	**Abnormal EMG**	**p value**
ODI	41.1 (9.3)	42.2 (9.4)	p = 0.280
VAS	31.5 (18.1)	41.9 (25.7)	p = 0.018
NRS leg pain	6.3 (2.1)	7.5 (1.5)	p = 0.000
NRS low back pain	4.2 (2.7)	4.8 (3.2)	p = 0.400
BDI	8.0 (3.9)	9.8 (3.8)	p = 0.014
Treadmill test	700 (426)	740 (385)	p = 0.642

Data are means, ± standard deviations in parentheses.

ODI = Oswestry disability index scale (0-100), VAS overall = Visual analogue pain scale (0-100), NRS LBP = NRS low back pain at rest, scale (0-10), NRS LP = NRS leg pain at walking, scale (0-10), BDI = Beck Depression inventory (0-63), treadmill exercise test (0-1000 metres).

### Correlation of MRI and EMG findings

Abnormal EMG correlated with the severity of mid zone stenosis in the visual assessment (p = 0.015). The entrance zone width was also somewhat lower in the roots with abnormal EMG (5.1 ± 1.7 mm vs 5.7 ± 1.9 mm; p = 0.050).

## Discussion

To the best of our knowledge, there are no previous studies investigating the severity of LLSCS by MRI and its associations with clinical symptoms and EMG findings. This study points out that there are associations between MRI-assessed LLSCS and abnormal EMG, indicating that LLSCS is a clinically significant finding. However, the severity of the LLSCS measured from MRI data did not correlate with the clinical symptoms. This may be explained by the small study sample but also by the multifactorial etiology of symptoms.

However, abnormal EMG findings were associated with the overall VAS, leg pain VAS and BDI, which suggests that EMG could be more sensitive than MRI for the detection of LLSCS.

In the context of previous publications [4-6], we did not find significant associations between the severity of stenosis in MRI and patient symptoms. However, we found associations between EMG and clinical symptoms. To the best of our knowledge there are no other prospective (LLSCS) studies evaluating the associations between EMG, MRI findings and clinical symptoms.

The strengths of this study are prospective study setting, recording of symptoms with validated questionnaires and tests, detailed visual and quantitative MRI analysis with confirmed reproducibility.

The results of the current study relate to routine clinical MRI with patients lying in the supine position. Imaging patients in the supine position is a limitation because patient symptoms may worsen in the upright position and the upright position may also alter the anatomy of the neural canal. Accordingly, the upright position would be the most appropriate image acquisition position to link image findings to patient symptoms [19-21]. Hiwatashi and colleagues found that axial loading with imaging can even influence treatment decisions [22]. Another limitation of this study is the small sample size; however, it is still large enough to provide the main results.

Lateral canal is a relatively small structure and use of 4 mm thick transversal slices, that were used to analyze subarticular zone, is suboptimal to visualize nerve roots accurately. It should be noted that at the subarticular zone the course on nerve root may be oblique to slice orientation and thinner slice thickness would provide higher accuracy especially for quantitative measurements. Moreover, even though we characterized some nerve roots to be compressed, we were not able to detect anatomical changes in nerve roots that would confirm the presence of true compression [23]. In optimal condition high resolution imaging with isotropic voxels conjoined with functional provocations could possibly improve diagnostic accuracy. Unfortunately such methods are not available for clinical routine. However, 3D isotropic SPACE sequence is currently available but it has not yet shown to improve diagnostic accuracy compared to 2D imaging [24].

We did not use paraspinal mapping technigue for EMG analysis which can be as a methodology weakness [4]. However our EMG analysis criterions are precise and the results are considered comparable with mapping technique.

Incidence of lumbar spinal stenosis is increasing due to the aging population [25,26]. Lumbar stenosis is detected also more frequently these days, due to the better quality and availability of radiological imaging facilities. These factors also increase the number of LSS operations. However, the selection of patients for surgical treatment still remains challenging. Our result strengthens the classical perception that the diagnosis of this syndrome arises from clinical history and radiographic evidence of a demonstrable stenosis [27-29].

The present study adds to the current knowledge by showing that EMG findings correlate with clinical symptoms in patients with LLSCS. This study also supports both the clinical use and research use of quantitative radiological classification of lateral spinal stenosis.

## Conclusions

Conclusions: Among persons previously selected for surgery, lateral stenosis seen on MRI correlates with EMG, and thus may be a clinically significant finding. Our EMG findings were also associated with patient symptoms. However, no relationships between the MRI findings and symptoms or walking capacity were found, suggesting their multifactorial etiology. MRI and EMG may be useful in the workup of patients with suspected LLSCS.

## Competing interests

The authors declare that they have no competing interests.

## Authors’ contributions

Guarantors of integrity for the entire study, OA, HK; study concepts/study design or data acquisition or data analysis/interpretation, all authors; manuscript drafting or manuscript revision for important intellectual content, all authors; approval of the final version of the submitted manuscript, all authors; literature research, PK, PS, VL, TA; clinical studies, TA, SS, TS, SM; statistical analysis, PK, VL, PS; and manuscript editing, all authors. All authors read and approved the final manuscript.

## Pre-publication history

The pre-publication history for this paper can be accessed here:

http://www.biomedcentral.com/1471-2474/15/247/prepub
